# Pembrolizumab-Induced Aplastic Anemia Successfully Treated With Thrombopoietin Receptor Agonist in a Patient With Lung Adenocarcinoma: A Case Report and Literature Review

**DOI:** 10.7759/cureus.98931

**Published:** 2025-12-10

**Authors:** Kensuke Namba, Kazutoshi Isobe, Sanshiro Nakao, Nobuyuki Hiruta, Hiroki Wakabayashi

**Affiliations:** 1 Division of Respiratory Medicine, Department of Internal Medicine, Toho University Sakura Medical Center, Sakura, JPN; 2 Division of Hematology, Department of Internal Medicine, Toho University Sakura Medical Center, Sakura, JPN; 3 Division of Pathology, Toho University Sakura Medical Center, Sakura, JPN

**Keywords:** aplastic anemia, eltrombopag, immune-related adverse events, pembrolizumab, thrombopoetin receptor agonist

## Abstract

A 71-year-old female with postoperative recurrence of lung adenocarcinoma was treated with chemotherapy plus immunotherapy (carboplatin, pemetrexed, and pembrolizumab). On day eight after the first cycle, she presented with fever and loss of appetite. Laboratory tests showed severe pancytopenia. Bone marrow biopsy revealed hypocellular marrow consistent with aplastic anemia (AA), which is considered an immune-related adverse event (irAE). Treatment with pulse glucocorticoid (GC) therapy, granulocyte colony-stimulating factor, and cyclosporine had a limited effect. On day 24, eltrombopag, a thrombopoietin receptor agonist, was started, subsequently leading to gradual hematologic recovery and discharge on day 34. IrAE-induced AA has a poor prognosis, with no established standard treatment. This case demonstrates the potential of thrombopoietin receptor agonists as a therapeutic option in GC-resistant irAE-induced AA.

## Introduction

Pembrolizumab, a humanized antibody that targets programmed cell death (PD)-1, has been increasingly used as an immune checkpoint inhibitor (ICI) for advanced malignancies, including non-small-cell lung cancer (NSCLC). Along with its use expansion, there has been an increase in immune-related adverse events (irAEs) that can affect multiple organ systems, including the lungs, gastrointestinal tract, and endocrine glands. Hematologic irAEs are rare but serious complications. For instance, aplastic anemia (AA) has a reported mortality rate of 54% with no established standard treatment [1]. AA is a disorder caused by various pathophysiological mechanisms leading to bone marrow failure to produce blood cells. IrAE-related AA is presumed to result from mechanisms such as enhanced cytotoxic T-cell activity and B-cell-mediated autoantibody production induced by anti-PD-1 therapy, leading to destruction of hematopoietic cells and suppression of bone marrow function [2]. This report discusses a case of glucocorticoid (GC)-resistant irAE-induced AA, which was successfully treated with a thrombopoietin receptor agonist. This article was previously presented as a meeting abstract at the Kanto regional meeting of the Japan Lung Cancer Society on March 8, 2025.

## Case presentation

**Figure 1:**
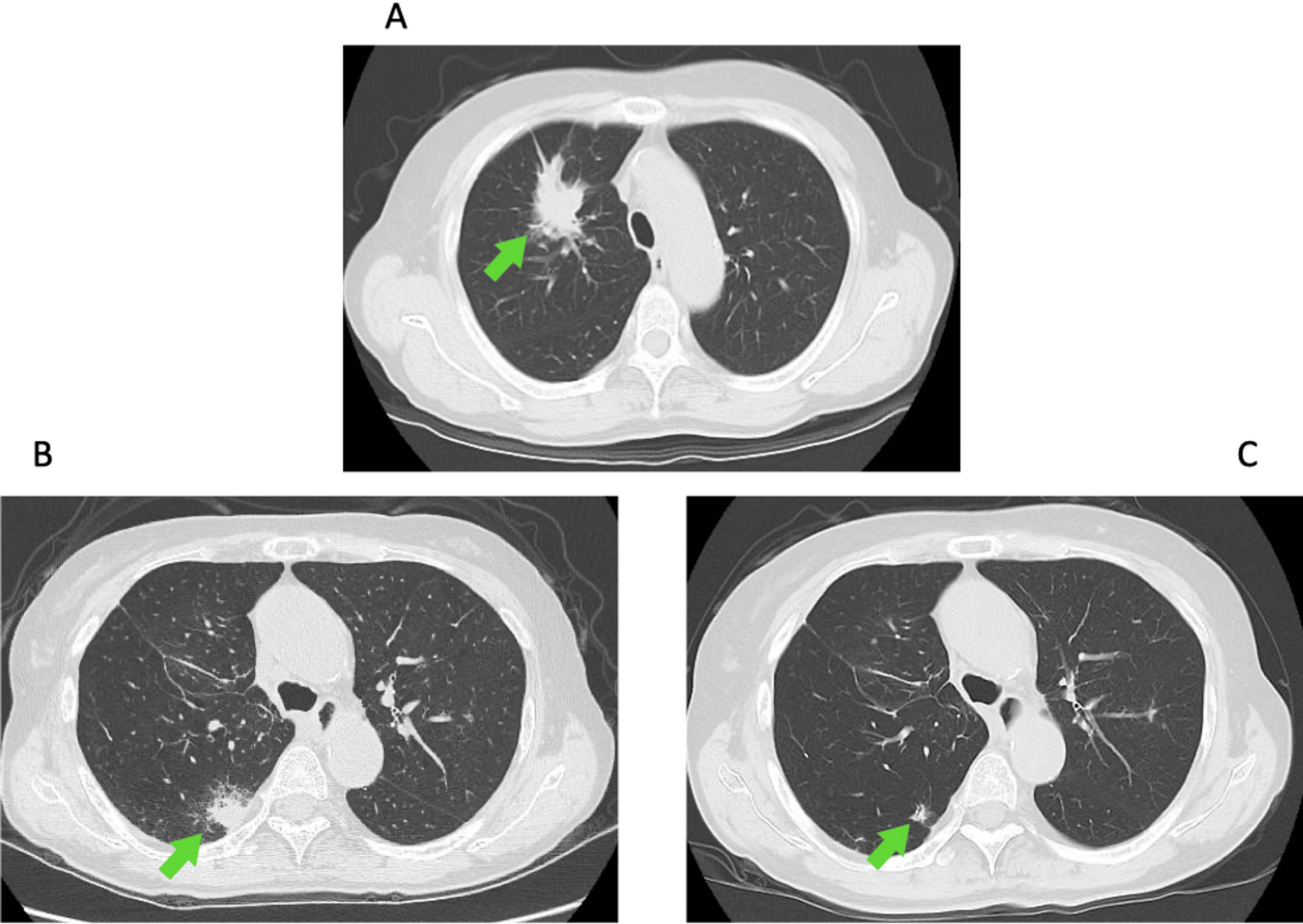
Representative CT images. A: Chest CT image pre-surgery reveals a mass shadow (arrow) in the right lower lobe.  B: Chest CT image at 14 months post-surgery reveals a recurrent nodule (arrow) in the right lower lobe. C: Chest CT image at 6 months post-chemotherapy initiation depicts a smaller nodular shadow (arrow).

A 71-year-old woman was referred to our hospital in February 2024 due to a right upper lobe lung mass incidentally found on chest CT (Figure 1A). This was diagnosed as lung adenocarcinoma (pT3N1M0, Stage IIIA), which was treated with right upper lobectomy and lymph node dissection, followed by two cycles of adjuvant chemotherapy with cisplatin and vinorelbine. The following year, a new nodule was detected in the right lower lobe (Figure 1B), suggesting recurrence. However, additional surgical resection and radiation therapy were not pursued due to severe obstructive ventilatory impairment caused by chronic obstructive pulmonary disease. Instead, combination therapy with pembrolizumab (200 mg/body), carboplatin (area under the blood concentration time curve = 5), and pemetrexed (500 mg/m²) was initiated. Pegfilgrastim (3.6 mg) was given prophylactically on day two.

**Figure 2:**
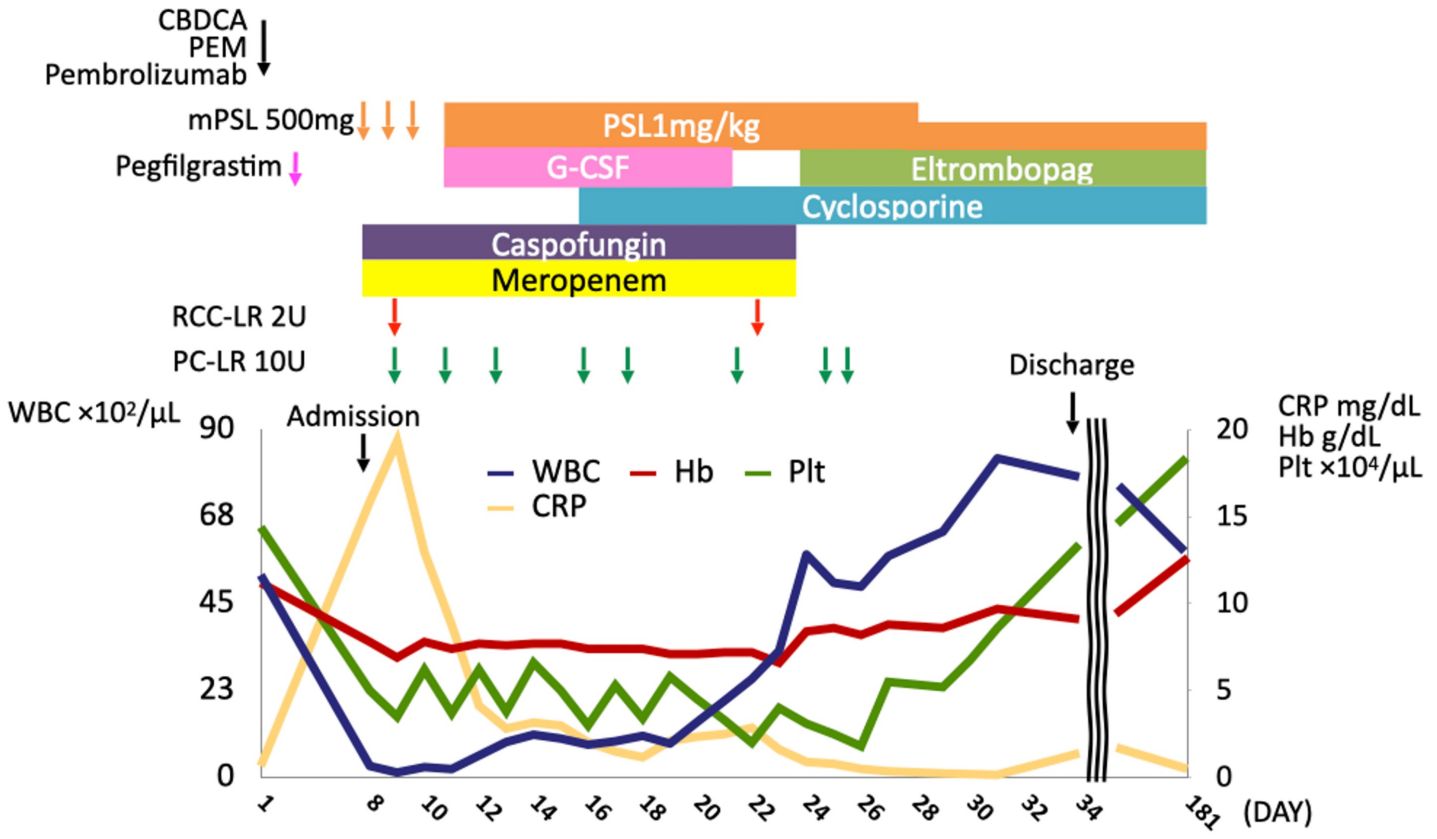
Clinical course. CBDCA: carboplatin; PEM: pemetrexed; mPSL: methylprednisolone; PSL: prednisolone; G-CSF: granulocyte-colony stimulating factor; RCC-LR: red cell concentrates-leukocytes reduced; PC-LR: platelet concentrates-leukocytes reduced; WBC: white blood cell; Hb: hemoglobin; Plt: platelet; CRP: C-reactive protein

On day eight of the first cycle of chemotherapy plus immunotherapy, the patient presented at our hospital with fever and anorexia. The clinical course is shown in Figure 2. Her medical history included left breast cancer, hypertension, and dyslipidemia, and ongoing hormone therapy with letrozole for breast cancer. She had a smoking history of 33-pack-years. Upon admission, she had a fever of 38.5°C, conjunctival pallor, and systemic erythema with oral mucosal lesions. Laboratory tests revealed pancytopenia (white blood cell count, 300/µL; red blood cell count, 206 × 10⁴/µL; platelet count, 5.0 × 10⁴/µL), elevated C-reactive protein (CRP), and liver and kidney dysfunction, whereas tumor markers were within normal range. A hematologic irAE was suspected due to the rapid progression of pancytopenia and concurrent systemic erythema diagnosed by a dermatologist as an irAE-related skin disorder, prompting pulse GC therapy and a bone marrow aspiration and biopsy.

Due to the rapid progression of pancytopenia and concomitant systemic erythema diagnosed by a dermatologist as an irAE-related skin disorder, pulse GC therapy and bone marrow aspiration and biopsy were performed.

**Figure 3:**
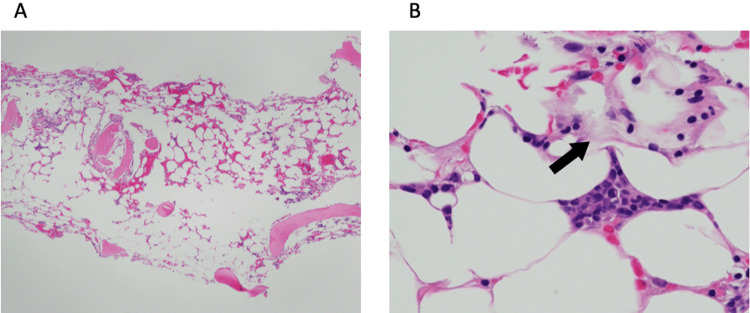
Microscopic histopathology. A: Hematoxylin-eosin staining at low magnification of bone marrow reveals severely hypocellular marrow with a low cellularity percentage. B: Hematoxylin-eosin staining at high magnification of bone marrow reveals gelatinous marrow transformation (arrow).

Bone marrow examination showed a marked decrease in nucleated cells and megakaryocytes. On biopsy, the marrow was severely hypocellular with a low cellularity percentage (Figure 3A), consistent with AA. Mild gelatinous marrow changes, admixed with preserved hematopoietic areas, were also observed (Figure 3B), suggestive of rapid marrow hypoplasia shortly before biopsy. Based on these findings and the rapid course of pancytopenia, irAE-induced AA was diagnosed.

Initially, the patient was treated with GC, filgrastim (150 mg/day), meropenem, caspofungin, and blood transfusions. The CRP improved rapidly, but the pancytopenia persisted despite administration of filgrastim. This course was considered atypical for chemotherapy-induced myelosuppression. On day 16 of chemotherapy plus immunotherapy, cyclosporine (CsA) was initiated for the treatment of AA. The white blood cell count improved to 5,770/μL on day 24, but the thrombocytopenia persisted at 3.1 × 10⁴/μL. Eltrombopag (EPAG), a thrombopoietin receptor agonist, was added on day 24. The platelet count gradually improved to 13.4 × 10⁴/μL, and the patient was discharged on day 34. Due to the severe irAE, only one cycle of chemotherapy plus immunotherapy was administered. Nevertheless, the patient maintained a partial response (Figure 1C) and continues regular outpatient follow-up at our hospital, with sustained normalization of all three blood cell lineages.

## Discussion

Hematologic irAEs occur in 0.5%-3% of patients receiving ICIs, with AA accounting for 6% of these [1]. ICI-induced AA has a high mortality rate, reaching up to 54% [3], and no standard treatment has been established. In this case, the initial treatment with GC and CsA was ineffective, but the thrombocytopenia improved after treatment with EPAG.

Thrombopoietin receptor agonists bind to the thrombopoietin receptor and trigger conformational changes that activate key intracellular signaling pathways, including the Janus kinase/signal transducer and activator of transcription and mitogen-activated protein kinase pathways. These cascades promote the proliferation and differentiation of megakaryocyte progenitors, leading to enhanced hematopoiesis and platelet production. In a phase 3 randomized controlled trial, adding EPAG to a regimen of antithymocyte globulin (ATG) plus CsA significantly improved outcomes, with overall response rates of 22% at three months and 68% at six months [4]. The current British Society for Haematology guidelines recommend EPAG as a second-line therapy for AA, following failure of anabolic steroids among patients with mild-to-moderate disease who are resistant to CsA [5]. EPAG is also recommended as an adjunct to CsA and ATG therapy in severe cases when transplantation is not feasible.

Including the present case, only 15 cases of irAE-induced AA have been reported to date (Table 1) [1,6-17]. The median age at onset of irAE-induced AA was 70 years, with a slight female predominance. NSCLC was the most common underlying malignancy, and nivolumab was the most frequently associated ICI. The median number of ICI cycles before AA onset was 4, although onset ranged from 1 to 12, indicating that AA can occur at any point during treatment and requires ongoing vigilance. Although standard immunosuppressive therapy for idiopathic AA achieves hematologic responses in 60%-70% of patients, with favorable long-term outcomes, the prognosis of irAE-induced AA is significantly worse [18]. Among the 15 reported cases, nine patients died, with six deaths directly attributed to AA. These deaths often occurred within two weeks to two months, suggesting a more aggressive course than idiopathic AA. EPAG was used in seven cases, but only the present case achieved hematologic recovery. In one case, because pancytopenia gradually worsened, it was difficult to distinguish from chemotherapy-induced myelosuppression. As a result, the diagnosis of AA and initiation of EPAG were delayed until 10 weeks after onset, which may have contributed to the poor prognosis. In this case, early immunosuppressive therapy is considered to have led to hematologic recovery.

**Table 1:** Cases of immune-related adverse event-induced aplastic anemia. ICI: immune checkpoint inhibitor; F: female; M: male; NSCLC: non-small cell carcinoma; AML: acute myeloid leukemia; RCC: renal cell carcinoma; IVIg: intravenous immunoglobulin; PSL: prednisolone; DEX: dexamethasone; mPSL: methylprednisolone; EPAG: eltrombopag; CsA: cyclosporin; ATG: antithymocyte globulin; GC: glucocorticoid; AA: aplastic anemia

Case	Age	Sex	Type of cancer	ICI	Cycle of ICI	Treatment	Outcome	Reference
1	73	F	NSCLC	Nivolumab	12	IVIg	No response to IVIg; death at 1 month from febrile neutropenia	Michot et al. [7]
2	70	M	NSCLC	Nivolumab	10	PSL 1 mg/kg, norethandrolone	Partial and transient response to GC; persistent pancytopenia still ongoing at 4 months	Michot et al. [7]
3	78	M	NSCLC	Nivolumab	1	PSL 1 mg/kg, IVIg	No response to GC and IVIg; death at 3 months from acute coronary syndrome	Michot et al. [7]
4	63	F	NSCLC	Nivolumab	2	DEX 12 mg	No response to GC; death at 2 weeks from complications of pneumonia	Filetti et al. [8]
5	74	F	Melanoma	Nivolumab	4	PSL 1.5 mg/kg, EPAG	No response to GC; death from AA	Rouvinov et al. [9]
6	61	M	AML	Nivolumab	11	mPSL 2 mg/kg	Good response to GC; complete remission was achieved	Cheng et al. [10]
7	57	F	Glioblastoma	Nivolumab	2	EPAG	Death at 73 days after the second dose of nivolumab from AA	Comito et al. [11]
8	72	F	NSCLC	Nivolumab	5	PSL 1 mg/kg, CsA, EPAG	No response to GC and immunosuppressive therapy; death from shock	Ghanem et al. [1]
9	67	M	Melanoma	Pembrolizumab	8	PSL 1 mg/kg, IVIg	Good response to GC; recovered 2 months later	Ni et al. [12]
10	77	F	NSCLC	Pembrolizumab	1	PSL 1 mg/kg, EPAG, danazol	Good response to danazol; death at 3 months from cancer	Goda et al. [13]
11	48	F	Melanoma	Ipilimumab/Nivolumab	5	PSL 1 mg/kg	No response to GC; death on day 11 of hospitalization from intracerebral hemorrhage	Helgadottir et al. [14]
12	51	M	Melanoma	Ipilimumab/Nivolumab	2	mPSL 1 mg/kg	Good response to GC; complete remission was achieved	Meyers et al. [15]
13	45	M	RCC	Ipilimumab/Nivolumab	2	PSL 1 mg/kg, IVIg, ATG, CsA, EPAG	No response to GC and immunosuppressive therapy; death at 2 weeks from AA	Younan et al. [16]
14	74	M	NSCLC	Pembrolizumab	6	CsA, EPAG, romiplostim, danazol	No response to immunosuppressive therapy; death at 14 months from cancer	Hashimoto et al. [17]
Present case	71	F	NSCLC	Pembrolizumab	1	Pulse glucocorticoid therapy, PSL 1 mg/kg, CsA, EPAG		

## Conclusions

We encountered a case of irAE-related AA that developed after pembrolizumab administration and responded favorably to EPAG. This case suggests the potential efficacy of EPAG for irAE-related AA; however, further case reports and clinical studies are needed to confirm this finding. Given that irAE-induced AA often progresses more rapidly and aggressively than idiopathic AA, early recognition and prompt treatment are essential. In patients receiving ICIs who develop pancytopenia, clinicians should maintain a high index of suspicion for hematologic irAEs and collaborate closely with hematologists to ensure timely and appropriate management.
